# Feasibility and acceptability of task sharing collection of HIV viral load dried blood spot samples with community lay cadres: A cross-sectional diagnostic validation study in Zimbabwe

**DOI:** 10.1371/journal.pgph.0006180

**Published:** 2026-03-31

**Authors:** Margaret L. Prust, Justin C. Graves, Juliet Jokwiro, Charity R. Giyava, Tsitsi Apollo, Raiva Simbi, Chiedza Mupanguri, Emmanuel Govha, Sandra Chipuka, Agnes Juru, Ashley Kallarakal, Nicole Kawaza, Yucheng Tsai, Tatenda Maparo

**Affiliations:** 1 Clinton Health Access Initiative, Inc., Boston, Massachusetts, United States of America; 2 Clinton Health Access Initiative, Inc., Harare, Zimbabwe; 3 Zimbabwe Ministry of Health and Child Care, Harare, Zimbabwe; 4 Clinton Health Access Initiative, Inc., Lusaka, Zambia; University of California San Francisco, UNITED STATES OF AMERICA

## Abstract

Viral load (VL) testing is a critical tool for clinical management of HIV, yet healthcare worker (HCW) shortages remain a key barrier to VL sample collection. This study assessed the feasibility and acceptability of VL dried blood spot (DBS) sample collection by community lay cadres (CLCs) compared to collection by trained HCWs. We implemented a cross-sectional diagnostic validation study across 10 purposefully selected public health facilities in Zimbabwe over six weeks in March-April 2024. Two DBS samples were collected from 374 participants: a reference sample collected by a HCW and a validation sample collected by a CLC. A subset of 173 CLC collections were observed using a checklist, and surveys were conducted with participating clients and CLCs. Diagnostic comparability was assessed using the proportion of matched pairs with agreement on viral load suppression status and Gwet’s AC1 statistic, while survey and observation data were analyzed using descriptive statistics. No samples were rejected by the laboratory, but two samples (one collected by a HCW and one by a CLC) were classified as invalid. Of the 372 paired tests analyzed, 96.0% (95%CI 93.2-97.7%) had concordant results, and the Gwet’s AC1 was 0.9564, indicating almost perfect agreement. All critical checklist items were done properly in 90.2% (95%CI 85.7-94.7%) of observed CLC collections. All CLCs reported confidence in performing DBS sample collection (89% very confident; 11% somewhat confident), and 94% of clients indicated willingness to have samples collected by a CLC in the future. These findings suggest that task-sharing VL DBS sample collection with CLCs is a feasible strategy, supported by strong diagnostic comparability, high CLC competency, and client acceptability. Policymakers should consider formalizing task shifting of VL sample collection within facilities. Further evidence on the feasibility of community-based DBS collection is needed.

## Introduction

Viral load (VL) is a critical indicator of the body’s response to antiretroviral therapy (ART) for human immunodeficiency virus (HIV) [1,2]. Since the World Health Organization (WHO) issued guidance in 2013 recommending VL as the preferred HIV monitoring method [3], extensive investments have been made in molecular laboratory systems for VL testing programs [4]. The availability of VL testing through clinic-based care has been an important contributor to reaching the estimated 93% of people living with HIV with suppressed VL among those on treatment globally [5]. The ability to obtain VL results from dried blood spot (DBS) samples and the emergence of point-of-care VL test processing has contributed to making this possible [6], Further enhancements in service delivery are required to ensure equitable progress, to reach the target of 95% viral suppression, and to strengthen the sustainability and success of long-term ART for both individuals and national programs.

In Zimbabwe in 2022, approximately 79% of the 1.18 million people on ART had had a VL test within the last year [7]. Challenges for VL coverage in Zimbabwe include health worker staff shortages to collect and process samples, poor coverage of clients in rural and hard-to-reach areas, long waiting times at facilities, availability of reagents, and VL funding issues [8,9].

Globally, HIV ribonucleic acid (RNA) levels in plasma samples are considered the gold standard globally for monitoring VL, but in settings where plasma tests or cold chain sample transport are not available, DBS tests are considered an acceptable alternative and a key tool for access [10,11]. Currently in Zimbabwe, VL testing is performed with both plasma and DBS samples, and samples are collected by trained health care workers (HCWs) [12].

Capacity for VL sample testing is currently centralized and concentrated in tertiary and provincial laboratories. Once VL samples are collected at health facilities, they are transported to laboratories via an integrated specimen transportation system. Sample registration and the identification and processing of urgent, point-of-care-eligible tests mainly occur at the district level, while testing other VL tests is conducted at provincial laboratories. Processed results are cascaded back to facilities electronically via SMS and/or email, and physical forms are returned via the specimen transportation system.

There are numerous bottlenecks and challenges that limit access to VL services, including overburdened facilities, HCW shortages leading to long wait times, an aging motorcycle fleet with frequent breakdowns, reagent stock outs, power instability in laboratories, inefficient data management systems, and poor linkages between laboratory and clinical systems [13].As differentiated service delivery (DSD) models expand, fewer patients are interacting with a HCW during routine visits [14].

Community health workers (CHWs) play a critical role in the delivery of a range of HIV services around the world, including low-resource settings [15]. The ability of CHWs, to establish trusting relationships with patients and provide a range of services has been noted as key factors for success [16]. In Zimbabwe, the term community lay cadres (CLCs) is used to refer to community health workers (called Village Health Workers in Zimbabwe), and peer-to-peer support mechanisms such as Community Adolescent Treatment Supporters (CATS), Community ART Refill Group leaders (CARGs), DSD group leaders, and expert clients. CLCs already interact with ART clients regularly, in the community and in facilities, and provide a range of services.

DBS samples are used for early infant diagnosis (EID) of HIV, and in other countries in Sub-Saharan Africa, EID DBS sample collection has been task-shared with lay cadres in order to overcome health workforce challenges [17]. Task sharing with lay cadres has also been successful for other blood-based tests, such as for malaria [18,19]. However, it has been noted that adequate training and assessment of skills is important to maintain quality of care in the context of task-shifting or task-sharing [20]. A small study from Zambia shows that non-medical personnel could feasibly collect DBS samples for viral load but the need for more data comparing test results from skilled and non-medical personnel was highlighted [21]. n Zimbabwe, community lay cadres (CLCs) play a crucial role in the provision of ART services. CLC is a term used to refer to community health workers (called Village Health Workers in Zimbabwe), and peer-to-peer support mechanisms such as Community Adolescent Treatment Supporters (CATS), Community ART Refill Group leaders (CARGs), DSD group leaders, and expert clients. CLCs already interact with ART clients regularly, in the community and in facilities, and provide a range of services. This study aimed to assess the feasibility and acceptability of CLC collection of DBS samples for VL testing as compared to DBS sample collection by trained HCWs.

## Methods

### Ethics statement

Ethical approval for this study was obtained from the Medical Research Council of Zimbabwe, Ref: MRCZ/A/3099.

### Study design

This cross-sectional diagnostic accuracy study included mixed methods components and was implemented in public sector health facilities in Zimbabwe. For this study, two DBS samples were collected from each participant, including a reference sample collected by a HCW and a validation sample collected by a CLC. A basic DBS test kit was developed by Lasec, including modifications such as self-retracting safety lancets and simplified packaging and instructions. Both samples were sent to the same laboratory for testing, but only the HCW-collected sample results were returned to the client. The laboratory staff were blinded to which cadre had collected each sample, and the test pairs were run on the same testing platform. A sub-sample of CLC collections were observed using a checklist, and surveys were also conducted with participating clients and CLCs. Recruitment took place from 13/03/2024 to 17/04/2024.

### Study sites and study population

This study was conducted in 10 public health facilities in Zimbabwe. We purposefully selected sites in close collaboration with Ministry of Health to include: primary care or level 1 hospital sites only; sites with at least 750 active ART clients; diversity in province, and representation that mirrors the national list of ART sites partner support and rural/urban location.

Within these 10 facilities, all ART clients who visited a study site during the study period (until the data collection sample size target was met) and were due for VL testing were invited to participate in the study. The intended sample size for the diagnostic accuracy component was at least 369 clients, based on the Donner and Eliasziw goodness-of-fit formula [22], which would allow 80% power and 95% confidence to detect a Cohen’s kappa or Gwet’s AC1 of 0.8 with a null value of 0.6, an expected proportion of suppressed clients of 90%, and a 10% buffer for invalid results or other sample processing issues not related to the sample collection comparison. We also estimated that a subset of at least 145 sample collections should be observed to estimate the proportion of CLCs accurately completing all steps with a 10% margin of error.

All CLCs engaged in HIV care from selected sites were eligible for inclusion. HCWs routinely collecting VL samples were eligible for inclusion as HCW sample collectors. Strict eligibility assessments were not conducted for CLC or HCW participants in order to simulate likely conditions for scale-up. Senior facility managers with clinical expertise were identified to be observers in each site.

### Data collection

CLCs received training in DBS sample collection, and HCWs from the study sites were trained to refresh their skills. A structured training program was implemented to ensure competency. CLCs participated in a 5-day training, of which a majority of time was spent covering HIV basics, the role of viral load monitoring in HIV patient management, patient interaction and other topics that the Ministry of Health aspires for CLCs providing HIV care to have competence in. Two days of this training covered DBS sample collection and related infection control measures. Before conducting sample collection, all CLCs demonstrated competency through supervised practice/mock collection, passing a skills assessment.

Following an informed consent process with clients agreeing to participate, DBS samples were collected by a CLC and then an HCW. For approximately every second CLC collection, an observer (the facility in-charge or laboratory staff) made observations using the checklist. After both samples were collected, the client was invited to participate in a survey. All participating CLCs were invited to complete a survey at the end of study. Written informed consent was sought from clients and CLCs prior to participation. Study staff entered survey and participant data into a study database using SurveyCTO (Dobility, Inc. Cambridge, MA, USA).

### Data analysis

All analyses were conducted in Stata SE 15 (StataCorp, College Station, TX, USA). Skills assessment and survey data were analyzed using descriptive statistics.

Diagnostic accuracy data was analyzed using Gwet’s AC1 and the percentage of matching pairs. Other statistics including sensitivity, specificity, Cohen’s kappa, the intraclass correlation coefficient (ICC), and McNemara’s test were explored and presented for triangulation and deeper understanding of the data. However, the nature and assumptions of these other statistics are less aligned the context of our study. Sensitivity and specificity are measures that require a comparison to a gold standard measure. In this case, the comparison test results are based on HCW-collected DBS samples, which cannot be considered as a gold standard because DBS does not have perfect accuracy as compared to plasma RNA [10,11,23]. In other words, it is possible that in cases where HCW- and CLC-collected samples disagree, either set of results could be incorrect. Cohen’s kappa is a statistical measure to assess the reliability between two raters by adjusting observed agreement beyond what would be expected by chance alone and is often used to assess agreement when neither measure is a gold standard [24]. However, Cohen’s kappa does not perform well and is subject to Kappa’s paradox where there is high prevalence of the outcome of interest, which we observed in the case of VL suppression [25–27]. Furthermore, the assumptions for Cohen’s kappa do not fully reflect our actual study conditions, suggesting this statistical approach may not be the best method to assess HCW and CLC comparability. Specifically, 1) the raters are not themselves assigning an outcome classification since this is done by the laboratory equipment conducting the DBS test, 2) nor are the raters independent from one another since the DBS tests are often run in the same lab by the same equipment, and 3) as the raters are not themselves assigning an outcome based on their judgement, this method isn’t useful in handling any change agreement or any underlying subjectivity that may exist [24]. In cases where key assumptions for Cohen’s kappa are not met or where Kappa’s paradox is present, experts have suggested the use of Gwet’s AC1 [28,29]. Then while McNemara’s test does not directly address inter-rater agreement directly, it would still be useful to compliment the analysis by assessing potential discordant classifications. Finally, the intraclass correlation coefficient (ICC) has also been used to compare two DBS samples [30,31], but our data do not meet core assumptions of an ICC analysis in that the data is not fully continuous and not normally distributed [32].

### Inclusivity in global research

Additional information regarding the ethical, cultural, and scientific considerations specific to inclusivity in global research is included in the Supporting Information (S1 Checklist).

## Results

### Study sample

A total of 375 clients consented to participate in this study (**Table 1**). One client withdrew from the study after the collection of one DBS sample, so samples from 374 clients were included in the study. Sixty CLCs, including primarily Village Health Workers (68%), were trained and enrolled to collect DBS samples. Fifty-eight HCWs participated in the study with 61% acting as DBS sample collectors and 38% as observers.

**Table 1:** Study participant characteristics.

Category	Characteristic	Number (%)
**ART Client Characteristics**		
*Total clients consented*		375 (100%)
*Gender*	Female	250 (66.7%)
*Age*	18-25 years	37 (9.9%)
	26-35 years	39 (10.4%)
	36-45 years	106 (28.3%)
	46-55 years	126 (33.6%)
	Greater than 55 years	67 (17.9%)
*Education*	No formal schooling	21 (5.6%)
	Some primary schooling	48 (12.8%)
	Completed primary school	122 (32.5%)
	Completed high school	171 (45.6%)
	Completed college or higher	13 (3.5%)
*Site location*	Rural	258 (68.8%)
	Urban	117 (31.2%)
*Site type*	Hospital	105 (28.0%)
	Primary care facility	270 (72.0%)
**CLC Characteristics**		
*Total CLCs enrolled*		60 (100%)
*Type of CLC*	Village Health Worker (VHW)	41 (68.3%)
	Community Adolescent Treatment Supporters (CATS)	9 (15.0%)
	Community Referral Facilitator (CRF)	3 (5.0%)
	Community Outreach Agent Coordinator (COAC)	2 (3.3%)
	Other	5 (8.3%)
*Average number of samples collected (among the CLCs that collected samples)*		7.2; min = 1, max = 18
*Number of trained CLCs that did not collect any samples*		8 (13.3%)
*Age* ^*1*^	21-35 years	5 (18%)
	36 to 45 years	11 (39%)
	46 years or greater	12 (43%)
*Gender* ^*1*^	Female	19 (68%)
*Years of experience* ^*1*^	2 to 3 years	6 (21%)
	4 to 7 years	5 (18%)
	8 to 12 years	9 (32%)
	Greater than 12 years	8 (29%)
**HCW Characteristics**		
*Total HCWs enrolled*		58 (100%)
*Study role*	DBS sample collector	36 (62.1%)
	Observer	22 (37.9%)
*Cadre*	Nurse	32 (55.2%)
	Sister in charge	16 (27.6%)
	Primary counsellor	6 (10.3%)
	Other	4 (6.9%)
*Average number of samples collected per sample collector*		10.7
*Average number of observations completed per observer*		7.5

HCW = Health care worker; CLC = Community Lay Cadre.

1. These CLC characteristics are based on the sample of 28 participants in the CLC survey.

### Diagnostic comparability and sample integrity

A total of 748 DBS samples were collected, and none were rejected by the laboratory. The results for two samples, each pertaining to separate clients, were classified as invalid; one was collected by a HCW and the other collected by a CLC. Both invalid results originated at the same hospital, were tested by the same laboratory, and were excluded from the analysis. This brought the final sample for diagnostic comparison to 372 unique clients, each with a paired set of VL results. DBS results were classified as suppressed if VL was below 1000 copies/mL and unsuppressed if VL was equal to or above 1000 copies/mL. In 96.0% (95% CI 93.2-97.7%) of paired results, the HCW- and CLC-collected samples had matching results (i.e., both designated as suppressed or unsuppressed).

Gwet’s AC1, the preferred measure to assess agreement in results given the study context, was 0.9564, which is categorized as “almost perfect” by Landis and Koch [33] (**Table 2**). Other measures of diagnostic accuracy are also included in Table 2. The sensitivity and specificity of CLC-collected samples compared to HCW-collected samples were 43.8% and 98.3%. Cohen’s kappa was 0.4620, representing a “moderate” level of agreement according to Landis and Koch [33]. The ICC for a two-way model of absolute agreement was 0.63, which is “good” agreement according to Cicchetti [34]. Finally McNemara’s test suggests there are no statistically significant differences in the classification between raters (χ² = 0.60, p = 0.44; exact p = 0.61), which further confirms high rater consistency without systematic misclassification.

**Table 2:** Diagnostic accuracy results comparing sample pairs collected by CLCs and HCWs.

	*Validation sample (CLC)*	
*Reference sample (HCW)*	Suppressed	Unsuppressed
**Suppressed**	350	6
**Unsuppressed**	9	7
Agreement (95% CI)	96.0% (93.4 – 97.7%)	
Gwet’s AC1	0.9564	
Sensitivity (95% CI)	43.8% (19.8 – 70.1%)	
Specificity (95% CI)	98.3% (96.4 – 99.4%)	
Positive predictive value	53.8% (25.1 – 80.8%)	
Negative predictive value	97.5% (95.3 – 98.8%)	
Cohen’s kappa	0.4620	
Intraclass correlation coefficient ^1^	0.63 (0.55 – 0.70)	
McNemara’s test	Χ²(1)= 0.60 (p = 0.44; exact p = 0.61)	

^1^Based on a two-way model of absolute agreement using average agreement for all DBS results.

### CLC skills assessment

The CLC sample collection was observed for a sub-sample of 173 clients (46.5%). Among the 52 CLCs who collected any DBS samples, 49 of them were observed at least once. The observation checklist included 23 items, of which 11 items were designated as critical. In 90.2% (95% CI 85.7-94.7%) of observations, all of the critical items were done properly. Observation scores were similar across CLC cadres and improved weekly over the six-week data collection period.

### CLC acceptability survey

Twenty-eight CLCs participated in an acceptability survey at the end of the study. Among them, 64% had never performed a blood-based test before, while others had done blood-based HIV or malaria testing. All participants reported a Likert scale degree of confidence; namely that they were very (89%) or somewhat (11%) confident that they could conduct DBS sample collection correctly. The survey asked about six steps of the sample collection process, and at least 93% or more of participants rated each step as very or somewhat easy (**Table 3**).

**Table 3:** CLC and client acceptability survey results.

Question/ category					Number (%)
**CLC background**					
*Ever performed blood-based tests prior to this pilot*					10 (36%)
***Ever trained in DBS prior to this pilot***					2 (7%)
**CLC experiences with DBS VL sample collection**					
*How would you rate your experience with…*					
	Very difficult	Somewhat difficult	Neither easy nor difficult	Somewhat easy	Very easy
**Finger prick**	0 (0%)	0 (0%)	2 (7%)	8 (29%)	18 (64%)
**Filling DBS spots on card**	0 (0%)	0 (0%)	2 (7%)	7 (25%)	19 (68%)
**Card drying**	0 (0%)	0 (0%)	0%	4 (14%)	24 (86%)
**Handling hazardous waste**	0 (0%)	0 (0%)	2 (7%)	2 (7%)	24 (86%)
**Sample collection instructions**	0 (0%)	0 (0%)	2 (7%)	4 (14%)	22 (79%)
**Sample collection overall**	0 (0%)	0 (0%)	2 (7%)	7 (25%)	19 (68%)
***How would you rate your confidence that you conducted sample collection correctly?***					
			Very unsure		0 (0%)
			Somewhat unsure		0 (0%)
			Neither sure or unsure		0 (0%)
			Somewhat confident		(11%)
			Very confident		(89%)
**Client preferences**					
***How comfortable did you feel when the community lay cadre collected blood samples from you?***			Not at all comfortable		6 (2%)
			A little bit comfortable		13 (4%)
			Neutral		19 (5%)
			Comfortable		157 (42%)
			Very comfortable		180 (48%)
*Would you do the DBS collection for HIV viral load testing by the community lay cadres again?*			No		15 (4%)
			Unsure		6 (2%)
			Yes		354 (94%)
*Would you do the DBS collection for HIV viral load testing by the community lay cadres again, and in a setting outside of the facility?*			No		45 (12%)
			Unsure		10 (3%)
			Yes		320 (86%)
*Would you recommend the DBS collection for HIV viral load testing to family members or friends by the community lay cadres?*			No		14 (4%)
			Unsure		12 (3%)
			Yes		349 (93%)
*What is your preference in the future for the approach for HIV viral load sample collection?*			Community (by CLCs)		181 (49%)
			Health facility (by HCWs)		96 (25%)
			No preference		98 (26%)

HCW = Health care worker; CLC = Community Lay Cadre.

### Client acceptability survey

A total of 375 clients participated in a brief survey after their DBS samples were collected. Clients provided generally positive feedback about CLC sample collection, with 90% feeling very comfortable or comfortable with CLC sample collection, and 94% indicating that they would do CLC sample collection again in the future (**Table 3**). Participants were asked about their preferences on the setting for VL sample collection, and 49% preferred community-based sample collection by a CLC, 25% preferred facility-based sample collection by a HCW, and 26% had no preference. Differences in client demographics were not associated with variations in preferences, but clients who received ART care at hospitals (as opposed to health centers) were significantly more likely to prefer community sample collection (**Table 4**).

**Table 4:** Percentage of clients by preference for future sample collection and demographics.

Category	Characteristic	In the community by CLCs	In the health facility by HCWs	No preference	p value
	Full sample	181 (49%)	96 (25%)	98 (26%)	
*Gender*	Male	66 (53%)	64 (26%)	71 (22%)	p = 0.321
	Female	115 (46%)	32 (26%)	27 (28%)	
*Age*	18-25 years	16 (43%)	15 (41%)	6 (16%)	p = 0.095
	26-35 years	14 (36%)	15 (38%)	10 (26%)	
	36-45 years	57 (54%)	24 (23%)	25 (24%)	
	46-55 years	58 (46%)	31 (25%)	37 (29%)	
	Greater than 55 years	36 (54%)	11 (16%)	20 (30%)	
*Education*	No formal schooling	13 (62%)	5 (24%)	3 (14%)	p = 0.806
	Some primary schooling	24 (50%)	15 (31%)	9 (19%)	
	Completed primary school	55 (45%)	33 (27%)	34 (28%)	
	Completed high school	82 (48%)	39 (23%)	50 (29%)	
	Completed college or higher	7 (57%)	4 (29%)	2 (14%)	
*Site location*	Rural	124 (48%)	75 (29%)	59 (23%)	p = 0.026
	Urban	57 (48%)	21 (18%)	39 (33%)	
*Site type*	Hospital	20 (60%)	19 (29%)	66 (12%)	p < 0.001
	Primary care facility	161 (19%)	77 (18%)	32 (63%)	

HCW = Health care worker; CLC = Community Lay Cadre. P values are based on chi-squared tests.

## Discussion

Task-shifting and task-sharing can be valuable strategies to relieve human resources and other constraints in a health system and to make services more accessible for clients, but it should not be undertaken without appropriate assessments of the quality and acceptability of care [35], and the need for this type of evidence guided our investigation.

This assessment found that CLC collection of DBS samples for HIV VL was highly acceptable and feasible. Observational assessments demonstrated the ability of CLCs to correctly and effectively use DBS VL sample collection kits. Surveys conducted with participating clients underscored that DBS VL sample collection by CLCs was a popular and welcome alternative to the standard HCW option. The diagnostic accuracy of paired samples of DBS tests was classified at “almost perfect” based on the Gwet’s AC1 test. Other measures of diagnostic accuracy were also explored and presented for this data, though our data does not fully meet the underlying assumptions required by those analytical approaches.

Task-sharing is an important strategy for addressing resource limitations and expanded access to care. This approach may be even more critical following donor funding cuts in 2025 that will impact HIV service delivery and health worker availability. As a result of expanding access to services, task-sharing can even lead to improved patient outcomes, such as in South Africa where point-of-care VL testing combined with task-shifting to junior level nurses improved viral suppression and retention in care [36]. The positive findings of this study about the appropriateness of task-sharing to CHWs or other lower-level cadres are corroborated by a number of others studies. Health Surveillance Assistants in Malawi, a low-level cadre of skilled personnel, and non-medical personnel in Zambia have demonstrated competency with collecting DBS samples for VL [21,37]. Patient-collected DBS samples have even been assessed and found to yield reliable results [38].

CHWs already play a crucial role in HIV service delivery in many countries. Especially in conditions of constrained resources, formal and informal task-sharing to CHWs is likely to be proposed. Task-sharing to CHWs presents various practical and ethical challenges related to the level of training, supervision and compensation that CHWs receive and the quality of the services they are capable of providing [39]. In considering efforts to task-share DBS sample collection with CHWs, assessments such as this one are required to ensure that the quality of care is maintained [20]. Additionally, literature suggests that attention must be given to establishing an enabling environment in which CHWs can be successful [40]. In this pilot for example, we included a five-day training for CLCs which covered HIV basics and other related topics not strictly required for the DBS sample collection task. Program managers considering task-sharing DBS sample collection with CHWs should consider the necessary foundational elements to set CHWs up well and may find efficiencies through integrating DBS training with other pre-service CHW training.

In recent years, HIV programs in high-burden settings have developed and scaled DSD models for individuals which reduce reliance on the facility [41]. As people visit health care facilities less frequently, access to appropriate laboratory testing, particularly VL, becomes less consistent. VL collection may be done on the same day as clients’ routine clinical visits and critical information around virologic response is not available until the next visit, which in some instances may be months away due to successes in multi-month refills.

Collection of VL samples in the community by CLCs could present an opportunity to innovate service delivery for decentralized and differentiated service delivery. Operational details of extending CLC sample collection into community settings need further investigation and piloting prior to deployment and implementation. Survey results from this assessment showed that some clients may prefer to continue receiving services at the facility for privacy or other reasons, but many would be open to VL sample collection in the community setting.

Even in the context of CLC sample collection for VL, the VL systems in Zimbabwe and other low resource settings still face a wide range of other challenges. These may include challenges related to sample transport and processing [42], long turn-around times [43], results return [44], and appropriate clinical action in response to VL results [45]. However, because sample collection is a first step in the VL cascade, addressing any barriers to sample collection, such as lack of human resources and long waiting times for sample collection, is an important priority.

This study has several limitations that should be considered in the interpretation of the results. As noted in the description of the methods, DBS does not represent a gold standard but was used as a comparison because it has been established as an adequate standard of care and would limit the risk and pain to participants. Both DBS samples were tested on the same laboratory platform, which introduced non-independence between paired results. Sites were selected purposefully with the goal of obtaining a diverse sample of sites that mirrored the national distribution of key characteristics of ART clinics, though the fact that the sample procedure was not strictly randomized may have introduced some level of selection bias. Client selection was based on convenient sampling and details about clients refusing were not systematically captured or analyzed. Finally, this study was conducted prior to substantial funding cuts to HIV programs that occurred in 2025, and funding cuts may have affected the staffing and resourcing of facilities in ways that could both increase the need for task-sharing while harming the enabling environment for service provision. Despite these limitations, the study provides important operational evidence on the feasibility and acceptability of task-sharing DBS collection to CLCs in routine program settings.

## Conclusions

This assessment suggests that task-sharing VL DBS sample collection with CLCs is feasible. Observational assessments showed that CLCs could correctly and effectively use DBS sample collection kits, and lab results from CLC- and HCW-collected samples were comparable. Client surveys confirmed strong acceptance of CLC-led collection. Policymakers in Zimbabwe and similar settings should consider updating policies and implementing task-sharing at the facility level. Further evidence is needed on the feasibility and acceptability of CLC-led DBS collection in community settings.

## Supporting information

S1 Checklist Inclusivity in global research questionnaire. (PDF)
