# Supplementary material for: Feasibility and acceptability of task sharing collection of HIV viral load dried blood spot samples with community lay cadres: A cross-sectional diagnostic validation study in Zimbabwe
Source: PLOS Glob Public Health. 2026 Mar 31;6(3):e0006180. doi: 10.1371/journal.pgph.0006180 (PMC13037983; doi:10.1371/journal.pgph.0006180)
Supplement: S1 Checklist — (PDF) [file pgph.0006180.s001.pdf]

Inclusivity in global research

PLOS' policy on inclusivity in global research aims to improve transparency in the reporting of research performed outside of researchers' own country or community and ensures that PLOS publications reporting global research adhere to high standards for research ethics and authorship. Authors of relevant research articles may be asked to complete the questionnaire below, which outlines ethical, cultural, and scientific considerations specific to inclusivity in global research. This questionnaire may be requested when researchers have travelled to a different country to conduct research, if research uses samples collected in another country, research with Indigenous populations or their lands, or if research is on cultural artefacts. Researchers travelling to another country solely to use laboratory equipment will not normally be required to complete the questionnaire. However, the questionnaire can be requested at the journal's discretion for any submission – if you have been requested to complete this questionnaire by the PLOS journal you submitted to, please do so.

Please complete the questionnaire below and include this as a Supporting Information file with your manuscript. Note that if your paper is accepted for publication, this checklist will be published with your article in the supporting information files. Please ensure that you reference the checklist in the main body of your manuscript. We suggest adding a subsection 'Inclusivity in global research' to your Methods section and adding the following sentence: "Additional information regarding the ethical, cultural, and scientific considerations specific to inclusivity in global research is included in the Supporting Information (SX Checklist)"

The questions have been designed to be applicable to a wide range of study types, and there are subsections for both human subjects research and non-human subjects research. If any of the questions are not relevant to your research please mark them as "N/A" as appropriate.

Ethical considerations, permits and authorship

This section is applicable to all research types.

Provide details as to who granted permissions and/or consent for the study to take place in the Methods section of your manuscript. This should include the names of **all** ethics boards, governmental organizations, community leaders or other bodies that provided approval for the study. If individuals provided approval refer to these people by their role or title but do not list their name(s).

Reported on page number: 5

Ethical approval for this study was obtained from the Medical Research Council of Zimbabwe, Ref: MRCZ/A/3099.

If there were any deviations from the study protocol after approval was obtained please provide details of these changes in the Methods section of your manuscript.

**N/A**

Did this study involve local collaborators that are residents of the country where the research was conducted or members of the community studied? If you do not have any authors from said communities, please provide an explanation for this below.

This work involved local collaborators that are residents of Zimbabwe where the research was conducted. The Zimbabwean collaborators that met the ICMJE criteria for authorship have been included as authors, including: Juliet Jokwiro, Charity R. Giyava, Tsitsi Apollo, Raiva Simbi, Chiedza Mupanguri, Emmanuel Govha, Sandra Chipuka, Agnes Juru, Nicole Kawaza, Tatenda Maparo.

Everyone listed as an author should meet PLOS' criteria for authorship and all individuals who meet these criteria should be included in the author byline, rather than the acknowledgements. For further information please see the journal's Authorship Policy.

Human subjects research (e.g. health research, medical research, cross-cultural psychology)

Did you obtain written informed consent from a representative of the local community or region before the research took place? How did you establish who speaks for the community? Details of written informed consent obtained from study participants should be reported separately in the Methods section of your manuscript.

The Ministry of Health and Child Care (MoHCC) is the custodian of health research conducted in Zimbabwe, and eEthical approval is the mandate of the Medical Research Council of Zimbabwe(MRCZ). After getting written approval from the (MRCZ), the research team obtained written approval to implement the research from MoHCC through the Permanent Secretary who is the Ministry's Chief Accounting Officer. In addition, written approval was obtained at subnational levels including the Provincial Medical Directorate, and District Medical Officers. From the initial conception, the study team engaged People Living with HIV (PLHIV). In particular, the Zimbabwe National Network of People Living with HIV (ZNNP+) was engaged to provide community presepctives because it has the widest representation across the country.

How did members of the local community provide input on the aims of the research investigation, its methodology, and its anticipated outcome(s)?

A co-creation process was used to develop the study protocol. This involved a series of consultative meetings with stakeholders across all levels, including national, sub-national, and community representatives. The Zimbabwe National Network of People Living with HIV (ZNNP+) served as the key community liaison, coordinating input from people living with HIV to ensure their perspectives were integrated. Following this inclusive process, a robust protocol was submitted to the Medical Research Council of Zimbabwe (MRCZ) for review and approval.

When engaging with the local community, how did you ensure that the informed consent documents and other materials could be understood by local stakeholders?

We employed a multi-faceted approach to ensure local stakeholders understood the consent materials:

- **Linguistic accessibility: Consent forms were translated into the relevant local languages to enhance comprehension for community lay cadres.**
- **Regulatory, contextual and cultural validation: The Medical Research Council of Zimbabwe (MRCZ) provided ethical approval, which includes a mandatory review to ensure all informed consent documents are appropriate for the local context and population.**

Will the findings of the research be made available in an understandable format to stakeholders in the community where the study was conducted (e.g. via a presentation, summary report, copies of publications, etc.)? Please provide details of how this will be achieved.

The study findings were disseminated to multiple audiences through tailored communication strategies to ensure comprehension. A primary dissemination meeting was held for district and facility-level staff, who were responsible for cascading the results to community lay cadres within their respective health facilities. To reach people living with HIV (PLHIV), the Zimbabwe National Network of People Living with HIV (ZNNP+), as the implementing partner further shared the findings within communities. Additionally, the results were presented at a Meaningful Involvement of People Living with HIV (MIPA) meeting to ensure direct engagement with PLHIV. All engagements were customized for their respective audiences, utilizing simplified messaging and vernacular language to foster clear understanding and uptake of the findings.

Non-human subjects research using specimens/ animals collected as part of the study, or those housed in archival collections. Examples include archaeology, paleontology, botany and zoology.

Did the permission you obtained from a local authority to perform the study include an agreement on access to outputs and benefit sharing? This may include procedures to enable fair distribution of the benefits and resources arising from the research performed. Please include any details of Prior Informed Consent and Benefit Sharing Agreements obtained. These may be required by field-specific regulations, for example the Convention on Biological Diversity (CBD) and the associated Nagoya Protocol.

N/A

If the material used in your study was imported, please A) provide the year it was imported and B) indicate whether permits were obtained to import/export the materials used, C) provide details of any permits obtained. If this information is not available, please indicate this.

For this study, DBS test kits were imported. The test kits had been adapted for community lay cadre use. The importation occurred in 2023 and we are submitting the rebate letter as supplemental documentation with the manuscript. Please indicate if any further documentation is required.

If you used archival specimens, please state how the material used in your study was acquired by the institute it is held in and provide details of any permits obtained for the original excavations/ sample collection. If this information is not available, please indicate this.

N/A

How was the potential cultural significance of the materials collected in your study to local communities considered in your research design? Were Indigenous peoples and/or local researchers and institutions involved with archaeological excavations / collection of specimens? If so, please provide a description of their involvement.

N/A

If your manuscript includes photographs of human remains please indicate whether authors obtained permission from descendants or affiliated cultural communities to do so.

N/A
